# Impact of clinical variables on outcomes in refractory cardiac arrest patients undergoing extracorporeal cardiopulmonary resuscitation

**DOI:** 10.3389/fcvm.2023.1315548

**Published:** 2024-01-05

**Authors:** Simon-Pierre Demers, Alexis Cournoyer, Olina Dagher, Pierre-Emmanuel Noly, Anique Ducharme, Hung Ly, Martin Albert, Karim Serri, Yiorgos Alexandros Cavayas, Walid Ben Ali, Yoan Lamarche

**Affiliations:** ^1^ Faculty of Medicine, Université de Montréal, Montreal, QC, Canada; ^2^ Department of Cardiology, Montreal Heart Institute, Montreal, QC, Canada; ^3^ Department of Emergency Medicine, Hôpital du Sacré-Cœur de Montréal, Montreal, QC, Canada; ^4^ Department of Surgery, Montreal Heart Institute, Montreal, QC, Canada; ^5^ Department of Cardiac Sciences, Libin Cardiovascular Institute, Calgary, AB, Canada; ^6^ Research Center, Montreal Heart Institute, Montreal, QC, Canada; ^7^ Department of Medicine, Critical Care, Hôpital du Sacré-Cœur de Montréal and CIUSSS NIM Research Center, Montreal, QC, Canada

**Keywords:** ECPR, cardiac arrest, extracorporeal circulation, cardiopulmonary resuscitation, cardiac intensive care

## Abstract

**Background:**

In the past two decades, extracorporeal resuscitation (ECPR) has been increasingly used in the management of refractory cardiac arrest (CA) patients. Decision algorithms have been used to guide the care such patients, but the effectiveness of such decision-making tools is not well described. The aim of this study was to compare the rate of survival with a good neurologic outcome of patients treated with ECPR meeting all criteria of a clinical decision-making tool for the initiation of ECPR to those for whom ECPR was implemented outside of the algorithm.

**Methods:**

All patients who underwent E-CPR between January 2014 and December 2021 at the Montreal Heart Institute were included in this retrospective analysis. We dichotomized the cohort according to adherence or non-adherence with the ECPR decision-making tool, which included the following criteria: age ≤65 years, initial shockable rhythm, no-flow time <5 min, serum lactate <13 mmol/L. Patients were included in the “IN” group when they met all criteria of the decision-making tool and in the “OUT” group when at least one criterion was not met.

**Main outcomes and measures:**

The primary outcome was survival with intact neurological status at 30 days, defined by a Cerebral Performance Category (CPC) Scale 1 and 2.

**Results:**

A total of 41 patients (IN group, *n* = 11; OUT group, *n* = 30) were included. A total of 4 (36%) patients met the primary outcome in the IN group and 7 (23%) in the OUT group [odds ratio (OR): 1.88 (95% CI, 0.42–8.34); *P* = 0.45]. However, survival with a favorable outcome decreased steadily with 2 or more deviations from the decision-making tool [2 deviations: 1 (11%); 3 deviations: 0 (0%)].

**Conclusion and relevance:**

Most patients supported with ECPR fell outside of the criteria encompassed in a clinical decision-making tool, which highlights the challenge of optimal selection of ECPR candidates. Survival rate with a good neurologic outcome did not differ between the IN and OUT groups. However, survival with favorable outcome decreased steadily after one deviation from the decision-making tool. More studies are needed to help select proper candidates with refractory CA patients for ECPR.

## Introduction

Cardiac arrest (CA) is a leading cause of mortality and affects approximately 550,000 patients in North America annually (1). In-hospital cardiac arrest (IHCA) treated with conventional cardio-pulmonary resuscitation (CPR) typically has a survival rate of 15%–17%, while out-of-hospital cardiac arrest (OHCA) survival is lower at 8%–10% (2, 3). Significant gains in survival rate have been reported in the past decades, attributable in part to early arrest recognition, bystander resuscitative efforts, early defibrillation, timely revascularization as well as advances of post-arrest care (4). However, despite adequate resuscitation maneuvers, 40%–60% of patients are unable to achieve return of spontaneous circulation (ROSC) and remain in a refractory CA state (usually defined as CPR duration of more than 20–30 min) (5, 6). The probability of survival with conventional CPR in such case is less than 5% (7).

In the past two decades, veno-arterial extracorporeal membrane oxygenation (VA-ECMO) during CPR, or extracorporeal resuscitation (ECPR), has been increasingly used in the management of refractory CA (8, 9). Extracorporeal resuscitation maintains organ perfusion while the underlying etiology of CA is determined and treated. ECPR use is growing rapidly with a reported annual rate from 247 cases in 2012 to more than 1,200 cases recorded in 2021 worldwide (10, 11). More recently, the Extracorporeal Life Support Organization (ELSO) Registry Report (April 2022) recorded a total of 11,761 cases of CA adults supported by ECPR with a 30% survival to hospital discharge (11). However, mortality varies significantly between centers, and many factors appear to critically determine ECPR success, notably patient selection (12). The 2021 ELSO consensus statement for ECPR also highlights that robust data to identify patients who may benefit from ECPR is lacking (13).

Decision algorithms have been used to guide the care of patients requiring emergent treatment suffering from a variety of conditions. These decision-making tools could allow team members to optimize the selection process for ECPR so that the best candidates can be cannulated as quickly as possible. As the initiation of ECPR ultimately relies on the decision of a multidisciplinary team, some patients are inevitably going to be cannulated outside of the criteria specified in such decision algorithms, either because of lacking information at the time of cannulation or because of the team clinical judgement. Different decision-making tools have been described in the literature (14), with different inclusion and exclusion clinical criteria. Such tools are being developed to improve decision-making effectiveness during refractory CA and to select patients with anticipated best chance of survival with ECPR. However, the effectiveness of such decision-making tools to appropriately select patients for ECPR has not been well described.

Our main objective was to compare the rate of survival with a good neurologic outcome of patients treated with ECPR meeting all criteria of a clinical decision-making tool for the initiation of ECPR to those for whom ECPR was implemented outside of the algorithm. Our secondary objectives were to explore the association between the number of deviations from the decision-making tool and outcomes of patients treated with ECPR and to describe the diagnostic value of each criterion of the decision-making tool.

## Materials and methods

### Study design and setting

This retrospective observational study was conducted using medical records of patients treated between January 2014 and December 2021 at the Montreal Heart Institute (MHI) in Montreal, Canada. The Institutional Review Board approved the study as posing minimal risk to patients, and it was therefore performed under a waiver of informed consent. The investigation conforms with the principles outlined in the World Medical Association Declaration of Helsinki.

The MHI is a tertiary academic center that offers cardiac interventional and surgical care with a 30-bed cardiosurgical Critical Care Unit. All options from temporary mechanical circulatory support to long-term ventricular assist devices and heart transplantation are available on-site. The Cardiohelp© device (Maquet Getinge Group, Wayne, NJ, USA) has been used for ECPR. In 2014, a decision-making tool was developed for refractory CA patients based on the current available literature, aiming to improve CA patients' outcomes. The tool was developed in collaboration with the institution's resuscitation committee.

In the case of a refractory arrest, the ECMO team (made of a cardiovascular surgeon, an interventional cardiologist and an intensivist) is activated through telephone notification. At the bedside, the team decides whether or not the patients is an appropriate candidate for ECPR based on the available cardiac arrest information and clinical variables. After getting the team's consensus, cannulation occurs through peripheral femoral cannulation on the site of arrest. For example, for an OHCA arriving in the emergency room (ER), cannulation occurs in the ER's resuscitation room. If the arrest occurs in the catheterization laboratory, cannulation occurs while perfoming PCI. Cannulation's guidance is provided by transoesphageal echocardiography and fluoroscopy, when available. For all cases, a mechanical compression device (LUCAS) was used during CPR. Upon cannulation, blood flows are set at 3.5–5.0 L/min in order to reach stable hemodynamics and regular aortic valve opening. Post-arrest targeted temperature management (TTM 33–36 C) was used in all patients.

In the case of left ventricular distension and loss of pulsatile flow, an algorithm for LV venting was used, aiming to help improve patients' outcomes as suggested by recent reports (15, 16). Optimal anticoagulation was achieved with unfractionated heparin. ECMO weaning was standardized for all patients (17).

### Study population

Adult patients (≥18 years old) who underwent ECPR during the 8-years study period were included in the present study. We dichotomized the patient cohort according to adherence or non-adherence with the ECPR decision-making tool, which included the following criteria: age ≤65 years, initial shockable rhythm, no-flow time < 5 min, serum lactate <13 mmol/L. Patients were included in the “IN” group when they met all criteria of the ECPR decision-making tool and in the “OUT” group when at least one criterion was not met. We did not study patients who experienced CA outside of ECPR cannulation team availability and patients with an underlying medical condition of poor prognosis (end-staged cancer, COPD on home oxygen, hemodialysis) or with an unwitnessed CA. These patients are systematically excluded for ECPR eligibility at our institution.

### Data collection

Data collection was conducted in a non-blinded fashion in adherence to recommended chart review methodology (18). The web-based Research Electronic Data Capture (REDCap) software was used for collecting our variables of interest. After extraction, the data was subsequently anonymized.

Baseline demographic and clinical characteristics, comorbidities, and CA etiologies were collected. If the CA was caused by an ischemic cardiac event, it was labeled as acute coronary syndrome. If no ischemic cause was found, the CA etiology was labeled as non-ischemic. Regarding the initial rhythm, we first analyzed this variable as dichotomic (shockable vs. non-shockable) but also performed analyses using it as an ordinal variable [shockable vs. pulseless electrical activity (PEA) vs. asystole]. For the pre-cannulation serum lactate, the worst value before ECPR initiation was collected. Neurologic outcomes were also ascertained through chart review.

### Outcome measures

The primary outcome measure was survival with favorable neurological status, defined as a Cerebral Performance Category (CPC) score of 1–2. The CPC score ranges from 1 (defined as conscious, alert, able to work), 2 (conscious, sufficient cerebral function for independent activities of daily life, able to work in sheltered environment), 3 (conscious, dependent on others for daily support), 4 (comatose, vegetative state) to 5 (brain death) (19). This variable was measured at 30 days or at hospital discharge if it occurred after 30 days of hospitalization, to ensure that all patients who were classified as having a favorable outcome survived their hospitalization.

### Statistical analysis

The entire eligible population was used. Descriptive statistics for the included cohort are presented using continuous variables as means with standard deviations or median with Q1–Q3, as appropriate, and categorical variables as frequencies with percentages. Comparisons between groups were performed using Mann–Whitney *U*-tests or Pearson's *χ*^2^ tests, as appropriate.

For the primary objective, the rate of survival with favorable neurological status was compared between both groups (IN and OUT) using a Fisher exact test. The number of patients meeting our primary outcome criteria was expected to be too low to perform adjusted analyses.

For the secondary objectives, we compared the rate of survival with favorable neurological status of patients treated with ECPR according to the number of deviations from the clinical decision-making tool also using a Fisher exact test, using pairwise comparisons. We then calculated the diagnostic value (sensitivity, specificity, positive and negative likelihood ratios) of each criterion of the clinical decision-making tool. Finally, we explored how our clinical decision-making tool could be optimized to differentiate patients with and without a favorable neurologic outcome.

All statistical analyses were performed using SPSS Statistics for Windows version 27.0 (IBM Corp, Armonk, NY, USA). The alpha level was fixed at 0.05.

## Results

### Baseline characteristics

**Figure 1:**
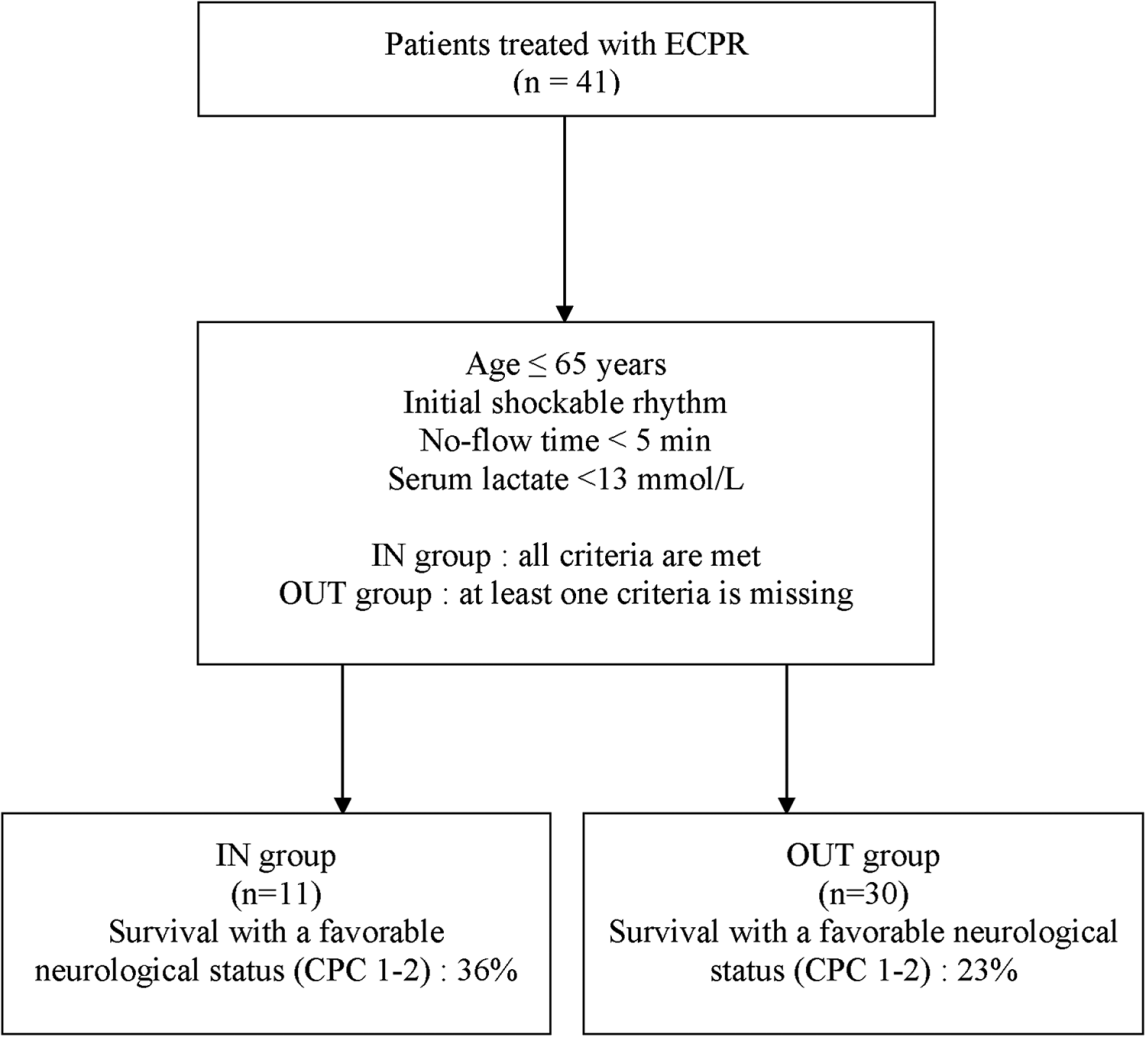
Study flow-chart; ECPR, extracorporeal resuscitation.

ECPR was performed on 41 patients between January 2014 and December 2021 at the MHI and were included in this study. Eleven (27%) patients received ECPR with adherence to all clinical variables in the ECPR decision-making tool (IN group) while 30 (73%) had at least one deviation from the local ECPR algorithm before cannulation (OUT group) (Figure 1). Nineteen (63%) patients had one deviation, 9 (30%) patients had 2 deviations and 2 (7%) patients had 3 deviations variables from the decision-making tool. The baseline demographic and clinical characteristics of all included patients are presented in Table 1. The most frequent variables of non-adherence were: serum lactate >13 mmol/L (17%, 57%), initial non-shockable rhythm (PEA: 11%, 37%; Asystole: 3%, 10%), age >65 years old (7%, 23%) and no-flow ≥5 min (3%, 10%). One patient did not have blood lactate measured before ECPR initiation but was classified in the IN group as he experience an IHCA in the catheterization laboratory and ECPR was implemented very rapidly (low-flow time = 20 min). As such, his lactates were measured only as ECPR was initiated, and were under 13 mmol/L. Baseline characteristics were relatively similar between both groups, apart from those who were used to classify patients, which were either statistically significant [Serum lactates <13 mmol/L: 10 (100%) vs. 13 (43%), *P* = 0.002; Initial shockable rhythm: 11 (100%) vs. 16 (53%), *P* = 0.005] or not statistically different [Age ≤65 years: 11 (100%) vs. 23 (77%), *P* = 0.16]. The most common arrest etiology in both groups was acute coronary syndrome [*n* = 29 (71%)], followed by non-ischemic [*n* = 10 (24%)]. ECPR was used more commonly in patients with IHCA (27%, 66%) than from OHCA (14%, 34%). The average ECMO run duration for the overall population was 5 ± 5 days. Six patients (15%) had withdrawal of life support within the first 24 h of cannulation.

**Table 1:** Baseline characteristics of patients, grouped according to the adherence to the decision-making tool.

	All patients (*n* = 41)	IN group (*n* = 11)	OUT group (*n* = 30)	*P*-value
Age, years	54 (11.0)	53 (10)	55 (12)	0.31
Age ≤65 years	34 (83)	11 (100)	23 (77)	0.16
Male	33 (80)	10 (91)	22 (73)	0.31
Body mass index	27.8 (5.3)	28.7 (4.6)	27.4 (5.6)	0.51
Comorbidities				
Diabetes mellitus	9 (22)	1 (9)	8 (27)	0.23
Hypertension	23 (56)	6 (55)	17 (57)	0.90
Dyslipidemia	21 (51)	6 (55)	15 (50)	0.80
Congestive heart failure	5 (12)	1 (9)	4 (13)	0.71
Coronary artery disease	16 (39)	5 (45)	11 (37)	0.61
Smoking	16 (39)	4 (36)	12 (40)	0.83
Chronic obstructive pulmonary disease	2 (5)	1 (9)	1 (3)	0.45
Chronic kidney disease	2 (5)	0 (0)	2 (6)	0.38
Location of arrest				0.64
Out-of-hospital cardiac arrest	14 (34)	5 (45)	9 (30)	
In-hospital cardiac arrest	27 (66)	6 (55)	21 (70)	
Arrest etiology				0.80
Acute coronary syndrome	29 (71)	9 (82)	20 (66)	
Non ischemic cardiomyopathy	10 (24)	2 (18)	8 (27)	
Myocarditis	8 (20)	1 (9)	7 (23)	
Acute on chronic heart failure	2 (4)	1 (9)	1 (3)	
Pulmonary embolism	1 (2)	0 (0)	1 (3)	
Intoxication	1 (2)	0 (0)	1 (3)	
Initial rhythm				0.015
Shockable rhythm	27 (66)	11 (100)	16 (53)	
Pulseless electrical activity	11 (27)	0 (0)	11 (37)	
Asystole	3 (7)	0 (0)	3 (10)	
**Initial shockable rhythm**	27 (66)	11 (100)	16 (53)	0.007
Bystander cardiopulmonary resuscitation	41 (100)	11 (100)	30 (100)	–
Immediate cardiopulmonary resuscitation	38 (93)	11 (100)	27 (90)	0.85
**No flow <5 min**	38 (93)	11 (100)	27 (90)	0.79
Time to cannulation, minutes	60 (40)	45 (25)	60 (43)	0.27
Low-flow ≤45 min	13 (32)	6 (55)	7 (23)	0.073
Pre-ECMO serum lactates, mmol/L	11.4 (4.5)	9.6 (2.5)	13.4 (4.7)	0.19
**Pre-ECMO serum lactates <13 mmol/L**	24 (59)	10 (100)	13 (43)	0.002
Location of ECMO cannulation				0.84
Emergency department	7 (17)	3 (27)	4 (27)	
Catheterization laboratory	26 (63)	8 (73)	18 (60)	
Intensive care unit	5 (12)	0 (0)	5 (16)	
Operating room	3 (7)	0 (0)	3 (10)	
SAVE score at admission, median (IQR)	−5.6 (4.1)	−3.9 (4.6)	−6.6 (3.7)	0.90

ECMO, extracorporeal membrane oxygenation; SAVE, survival after veno-arterial ECMO score.

Values are presented as median (interquartile range) or *N* (%); Clinical variables included in the decision-making tool are bolded.

### Main results

**Figure 2:**
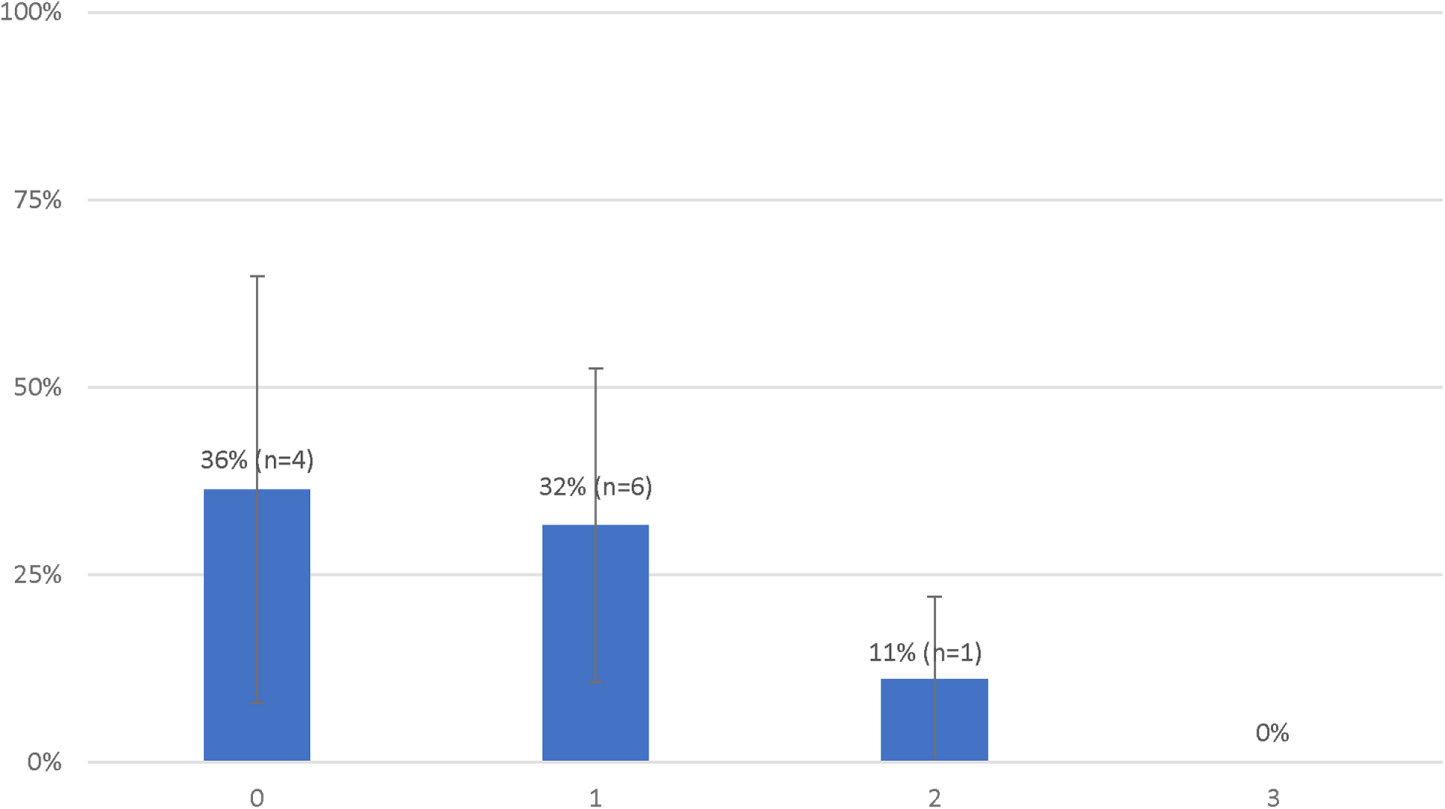
Probabilities of favorable neurological status of patients treated with extracorporeal resuscitation according to the number of deviations from the decision-making tool.

A total of 4 (36%) patients met the primary outcome in the IN group and 7 (23%) in the OUT group (Figure 2). There was no statistical difference in the rate of survival with a favorable neurologic outcome between both groups [odds ratio (OR): 1.88 (95% CI, 0.42–8.34); *P* = 0.45]. There were not enough events to perform a multivariable analysis.

The rate of survival with a favorable outcome according to the number of deviations from the clinical decision-making tool are presented in Figure 2. There was no statistical difference in the rate of survival with a favorable neurologic outcome between patients with 0 or 1 deviation from the clinical decision-making tool [4 (36%) vs. 6 (32%), *P* = 1.00]. Survival with a favorable outcome decreased with subsequent deviations [2 deviations: 1 (11%); 3 deviations: 0 (0%)] however, the difference between patients with 0 or 1 deviation as compared to those with 2 or more deviations [10 (33%) vs. 1 (9%), *P* = 0.23] was not statistically significant.

Table 2 illustrates the sensitivity and specificity of each individual variable of the decision-making tool. Notably, all patients with initial asystole (*n* = 3) and with a no-flow time of ≥5 (*n* = 5) minutes died (Sensitivity = 100%). Sixty-four percent (*n* = 7) of survivors had an initial shockable rhythm [Sensitivity 64%, specificity 33%, LR + 0.95 (0.57–1.60)]. The presence of a low-flow time of 4in or less was associated with favorable neurologic outcomes [OR = 7.00 (95% confidence interval 1.53–32.00), *P* = 0.12].

**Table 2:** Sensitivity, specificity, positive likelihood ratio and negative likelihood ratio of each criterion of the decision-making tool to identify patients who survived with a favorable neurologic outcome.

Variable	Survival with a favorable neurologic outcome (*n* = 11)	Death (*n* = 30)	OR (95% CI)	*P*-value	Sensitivity (95% CI)	Specificity (95% CI)	Positive likelihood ratio (95% CI)	Negative likelihood ratio (95% CI)
Age ≤65 years old	10 (91%)	24 (80%)	2.50 (0.27–23.53)	0.42	91% (57%–100%)	20% (8%–39%)	1.14 (0.87–1.47)	0.45 (0.05–3.83)
No-flow time <5 min	11 (100%)	25 (83%)	NA	NA	100% (68%–100%)	17% (6%–35%)	1.20 (1.02–1.41)	0 (0–NA)
Initial shockable rhythm	7 (64%)	20 (67%)	0.88 (0.21–3.71)	0.86	64% (32%–88%)	33% (18%–53%)	0.95 (0.57–1.60)	1.09 (0.44–2.72)
Initial lactate <13 mmol/L	8 (73%)	15 (52%)^a^	2.49 (0.55–11.31)	0.24	73% (39%–93%)	48% (30%–67%)	1.40 (0.85–2.32)	0.56 (0.20–1.59)
Initial rhythm ≠ asystole	11 (100%)	27 (90%)	NA	NA	100% (68%–100%)	10% (3%–28%)	1.11 (0.99–1.25)	0 (0-NA)
Low-flow time ≤45 min	7 (64%)	6 (20%)	7.00 (1.53–32.00)	0.012	64% (31%–89%)	80 (61%–92%)	3.18 (1.37–7.40)	0.45 (0.20–1.01)

95% CI, 95% confidence interval.

^a^ 1 patient with a missing value.

**Figure 3:**
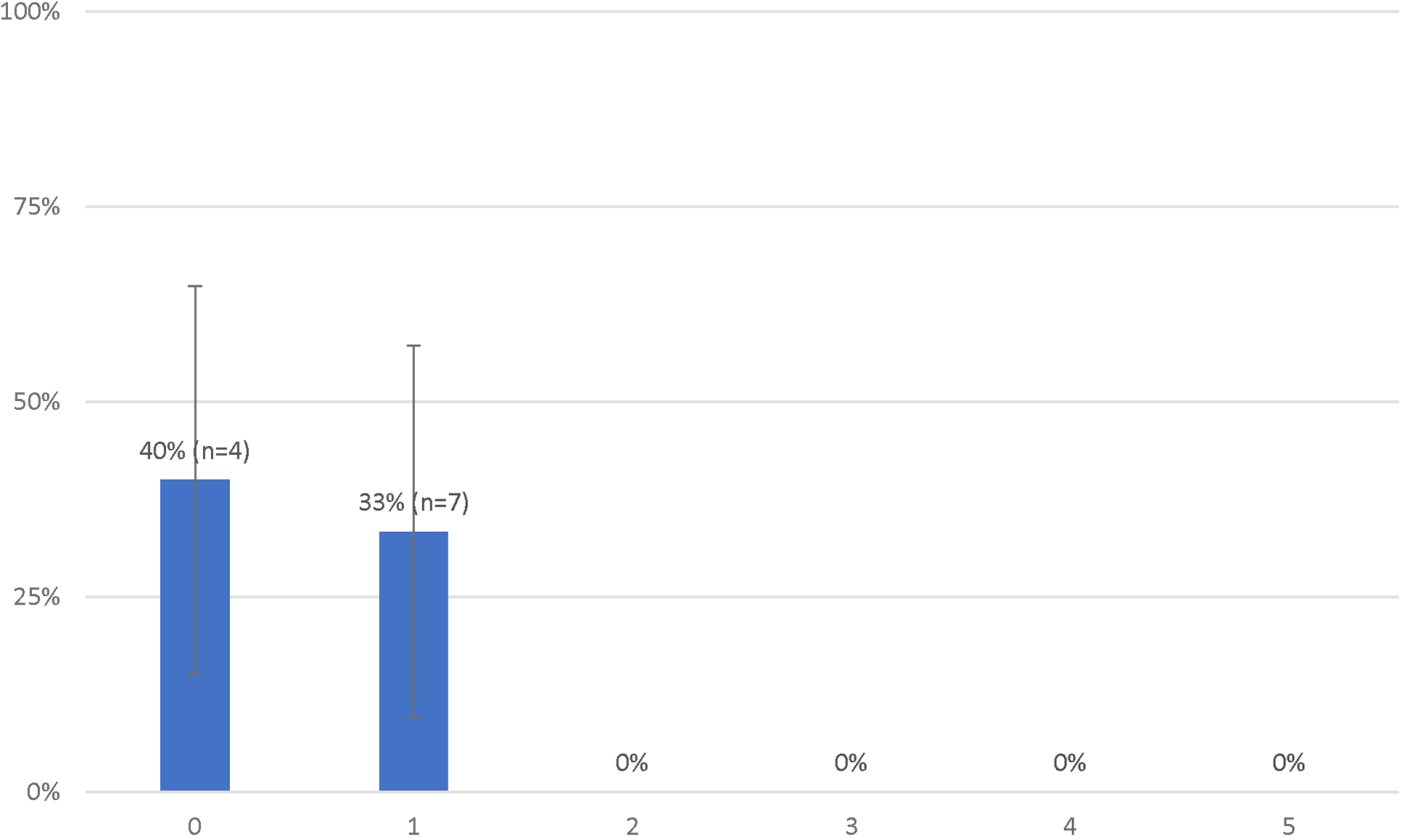
Probabilities of favorable neurological status of patients treated with extracorporeal resuscitation according to the number of deviations from the modified decision-making tool.

Considering these results, we performed an analysis in which we considered the initial rhythm as an ordinal criterion (Table 3). As such, the presence of an initial PEA added one deviation while an initial asystole added two deviations. In this analysis, the presence of a no-flow time ≥5 min also added two deviations and patients who had a low-flow time of 45 min or less were considered to have one less deviation (if they had one to begin with). The rates of survival with a favorable outcome according to the number of deviations from this modified decision-making tool are presented in Figure 3. Patients with 0 and 1 deviation were more likely to survive with a favorable neurologic outcome as compared to those with 2 deviations or more [11 (37%) vs. 0 (0%), *P* = 0.02].

**Table 3:** Modified decision-making tool for ECPR patients’ selection.

Clinical variables	Deviation points
Initial rhythm	
Asystole	2
PEA	1
No flow time ≥5 min	2
Low flow time ≤45 min	−1
Total	Survival
0 point	40%
1 point	33%
≥2 points	0%

## Discussion

In this cohort study of cardiac arrests patients receiving ECPR, we observed that survival with a good neurologic outcome did not differ significantly between patients selected within and outside the variables of a decision-making tool. However, patients' survival seemed to decrease rapidly when more than one deviation from the tool were present. We therefore modified our clinical decision-making tool to adequately discriminate between patients with and without good outcomes. Indeed, such decision-making tools are designed to guide the care of CA patients in identifying those optimal for ECPR implantation (20, 21). The criteria included in our clinical decision tool had, individually, a relatively poor predictive accuracy to identify patients with a good neurologic outcome. However, we observed that bundling individual criteria could help discriminate patients with a higher chance of survival, as demonstrated by the improved survival with good neurological outcome in our modified decision-making tool. These results reflect the complex interaction between individual factors and the clinical condition, suggesting they should be analyzed as a whole before ECPR implementation.

Regarding the cardiac rhythm at ECMO initiation, our finding partly contrasts with previously published studies, which showed that patients with a shockable rhythm had a 5–15 higher likelihood of survival compared to patients with non-shockable rhythms (22–24). A recent observational study by Pozzi et al. (25) also showed that adding sustained shockable rhythm as a strict criterion for ECMO implementation improved the survival to discharge with CPC 1–2 from 4% to 24% in their cohort. However, in a retrospective study, Yoshida et al. (26) observed that ECPR can be implemented in OHCA with non-shockable rhythms with a fair overall survival (23%) in carefully selected patients with a short low-flow time. Similarly, patients in our cohort implanted with non-shockable rhythms did not have other deviation from protocol, while patients with a shockable rhythm had more frequently other deviations from protocol. Nevertheless, the presence of asystole in our cohort universally identified patients with bad outcomes, suggesting that the presence or not of a shockable rhythm should be considered alongside the other clinical variables for ECPR candidacy selection.

The presence of a no-flow time of 5 min or more always identified patients who had worse outcomes, while a low-flow time of 45 min or less showed the best positive likelihood for good outcomes. These results are in line with several small studies (20, 27, 28) which have shown a rapid decline in survival with increased resuscitation duration. Indeed, no-flow duration over 5 min confers a dismal prognosis in cardiac arrest patients (29, 30). Low-flow duration should obviously be as short as possible and Otani et al. (27) suggested that 58 min was possibly the cut-off for optimal survival and Chen et al. (31) showed a significant increase in survival (OR, 9.8) with CPR duration of less than 60 min when compared to those above 1 h. This is obviously explained by patients with the shortest no- and low-flow duration having reduced time of hypoperfusion and ensuing suffering for the brain and the body. This emphasizes the importance of early ECMO-team activation in CA patients.

The presence of a lactate less than 13 mmol/L had an average sensitivity but a poor specificity to identify patients at good outcomes following ECPR. Different lactate thresholds (32–34) have been used for ECPR selection. In a retrospective nationwide OHCA study, Gregers et al. (33) observed that admission lactate lower than 13.2 mmol/L was associated with improved outcomes in patients treated with ECPR. Likewise, Halenarova and colleges (34) showed that pre-ECMO lactate values above 13.5 mmol/L in IHCA and above 16 mmol/L in OHCA were associated with worse prognosis, reflecting the greater metabolic derangement in these patients. Moreover, lactate clearance in the first hours after ECPR, and not only the initial absolute value, could help identify patients with a favorable prognosis (35). Therefore, although absolute lactate thresholds vary in the literature, limiting the degree of tissue hypoxia and hypoperfusion before ECPR implantation can help improve outcomes in this population.

Patient's age of less than 65 years had a good sensitivity but a poor specificity to identify patients at good outcomes following ECPR. Age has been reported as an unfavorable prognostic factor in ECPR patients, and different cut-offs have been proposed in the literature (36, 37). In a small retrospective study, Axtell et al. (38) observed that young age was the most important predictor of good neurological function, with a threshold at 60 years old. Other studies (39, 40) have shown that less than 3% of patients above 75 years old that received ECPR have a good neurologic outcome. Elderly patients have a lower cardiovascular and respiratory physiological reserve than youngers, which can explain their inability to withstand prolonged CA and ECPR.

From our results, we derived a modified clinical decision tool that improve discrimination between patients with and without good outcomes. In this algorithm, patients were still considered eligible with one deviation (or even two deviations if they had a low-flow time of less 45 min). As such, we probably need to have flexibility when implementing ECPR to ensure that we do not miss out on patients with a fair chance of good outcome. A future study should prospectively evaluate our modified algorithm and investigate how it can be combined with other clinical markers of cerebral blood flow, such as the presence of signs of life during resuscitation (41).

### Limitations

Our retrospective observational design comes with inherent limitations. Patients for whom ECPR was initiated outside of the clinical decision tool might have had some unmeasured characteristics which made them at better prognosis. However, we captured most known determinants of survival following ECPR and these were accounted for in our interpretations of the results. Our small sample size limit our ability to perform multivariable analyses and to assess complex interactions with confounders, which is a common issue in this particular field of study. Finally, our results might not be generalizable to other regions, as ECPR performance or CA patients' characteristics could differ. However, our observed overall outcomes (survival with good neurological outcome = 27%) are consistent with previous reports showing a survival rate between 23%–43%, which hints at similar patients and treatments (13–20, 21–42).

## Conclusion

Most patients supported with ECPR at our institution fell outside of the criteria encompassed in our clinical decision-making tool, but they did not differ in their survival rate with a good neurologic outcome of those who met all criteria. However, survival with favorable outcome decreased steadily after one deviation from the original decision-making tool. Using a modified algorithm, no patients with 2 or more deviations exhibited survival with favorable neurological status. Our results emphasize the need to improve selection when considering all patients with a fair chance of survival, for better outcomes while avoiding futility.

## Data Availability

The raw data supporting the conclusions of this article will be made available by the authors, without undue reservation.
